# Effects of nutrition education on weight gain prevention: a randomized controlled trial

**DOI:** 10.1186/s12937-016-0150-4

**Published:** 2016-03-31

**Authors:** Catherine J. Metzgar, Sharon M. Nickols-Richardson

**Affiliations:** Department of Food Science and Human Nutrition, University of Illinois, 282 Bevier Hall, 260A Bevier Hall, 905 S. Goodwin Ave, Urbana-Champaign, IL USA

**Keywords:** Body weight, Obesity, Overweight, Weight gain prevention, Weight loss, Weight management, Women

## Abstract

**Background:**

Body weight (BW) reduction through energy restriction is ineffective at impacting the obesity epidemic. Shifting from an obesity treatment to weight gain prevention focus may be more effective in decreasing the burden of adult obesity.

**Methods:**

This was a 1-year randomized controlled trial of weight gain prevention in healthy premenopausal women, aged 18–45 y, with a body mass index (BMI) of >18.5 kg/m^2^. Eighty-seven women were randomized to a weight gain prevention intervention delivered by a registered dietitian (RDG) or counselor (CSG), or to a control (CON) group. Eighty-one women (mean ± SD, age: 31.4 ± 8.1 y; BW: 76.1 ± 19.0 kg; BMI: 27.9 ± 6.8 kg/m^2^) completed baseline testing and were included in intention-to-treat analyses; anthropometric, blood pressure, dietary intake and physical activity measurements and biochemical markers of health were measured every three months. Data were analyzed using repeated measures ANCOVA, with significance at *P* < 0.01.

**Results:**

Sixty-two percent of women met the weight gain prevention criteria (BW change within ±3 %) after one year; this did not differ by group assignment. Body fat % was lower in the RDG versus CSG and CON groups at all intervals (*P* < 0.001). Systolic blood pressure increased from month 6 to 9 and decreased from month 6 to 12 in the CON group (*P* < 0.001), with a significant group x time interaction (*P* < 0.01). Estimated carbohydrate intake (%) was higher in the RDG vs. CON group at month 9 (*P* < 0.01); fat intake (%) was lower in the RDG vs. CON group and CSG vs. CON group at months 3 and 9, respectively (*P* < 0.01). Estimated fruit intake (svgs/d) was higher in the RDG vs. CON group at months 3, 6, 9 and 12 (*P* < 0.01), and non-meat protein sources (svgs/d) was higher in the RDG vs. CSG and CON groups at month 3 (*P* < 0.001). Estimated energy, macronutrient and food group intakes did not change over time.

**Conclusions:**

A majority of all participants maintained BW over one year and were able to do so regardless of whether they received nutrition education. Additional studies that include a variety of clinical outcomes are needed to evaluate further aspects of nutrition education on weight gain prevention and health status over the long term.

## Background

More than two-thirds of adults and nearly two-thirds of women in the United States remain overweight and obese, even as the prevalence of overweight and obesity has stabilized in recent years [1]. Energy restriction and other methods to induce body weight (BW) reduction are popular and widely promoted [2, 3]. While a variety of approaches result in short-term weight loss success [4–11], none of these methods have significantly impacted the obesity epidemic by permanently reducing BW over the long term for a significant amount of people [2, 4, 12–16]. In fact, among individuals who have achieved weight loss, most return to initial weight status within three to five years following weight loss [13–15], and one-third to two-thirds of these individuals will regain more weight than was initially lost [4]. Even individuals who undergo bariatric surgery gradually regain weight over time [17, 18]. Therefore, new prevention or treatment efforts and solutions to reduce the burden of adult obesity are necessary.

One such alternative may be shifting from a weight loss treatment approach to a weight gain prevention and health promotion approach. Weight gain prevention may also be referred to as weight maintenance and literally implies no change in BW. Unlike weight loss, which is relevant only for individuals with excess BW, weight gain prevention is applicable for individuals regardless of BW status. In individuals of normal weight, overweight or obesity, weight gain prevention can help manage current diseases and related risk factors [19], prevent the development of metabolic abnormalities and prevent the progression to overweight and/or obesity [20]. While other health indicators, such as blood pressure and blood lipid levels, may be improved with weight loss, these benefits may be mitigated with weight regain. However, positive behavior changes have been shown to result in similar improvements in blood pressure [20–24] and blood lipid levels [20, 21, 24–26], even in the absence of weight change.

Research investigating weight gain prevention interventions is limited [27–35], and few studies have found significant effects of interventions on preventing weight gain [27, 34]. Study populations have differed by gender (female only or males and females), BW status (normal weight only, normal and overweight, overweight and obese only), and intervention (newsletters, group education, individual counseling). As determinants of weight gain prevention may differ between men and women [3, 36–38], the current study aimed to examine weight gain prevention in premenopausal women participating in a 1-year randomized controlled trial of weight gain prevention that included nutrition education. Women randomized to nutrition education intervention groups were hypothesized to maintain current BW, within ±3 % [39], over the 1-year intervention period as compared to a control group. Further, it was hypothesized that women randomized to a nutrition education group led by registered dietitians would have lesser weight gain over the 1-year intervention period as compared to women randomized to an identical nutrition education group led by counselors with no formal nutrition training.

## Methods

### Participants

Premenopausal women with a body mass index (BMI) of >18.5 kg/m^2^ and aged 18–45 years were recruited from the Urbana-Champaign communities and surrounding areas of Illinois. Full details regarding recruitment, screening and enrollment have been previously described along with complete inclusion and exclusion criteria [40]. Briefly, women were eligible to participate if they met age and BMI criteria and desired to prevent weight gain. Women were excluded if they were amenorrheic; presented with depressive symptomology as suggested by a score of >50 on the Zung Self-Rating Depression Scale/Status Inventory [41]; self-reported cardiovascular, metabolic or musculoskeletal abnormalities or used medications to manage such conditions; used supplements and/or medications that may influence BW regulation; had undergone weight loss surgery; or were currently pregnant, lactating or planning to become pregnant.

The Institutional Review Board (IRB) for the Protection of Human Subjects at the University of Illinois at Urbana-Champaign approved the study protocol (IRB#14397). Each participant provided written informed consent prior to participating in the study.

### Study design and intervention

This was a 1-year parallel-arm randomized controlled trial of weight gain prevention that was conducted between August 2014-August 2015. The definition of within ±3 % change in BW from baseline, proposed by Stevens and colleagues [39], was used as the *a priori* criterion for weight gain prevention, with change of > ±3 % in BW as non-weight maintenance. After enrollment, participants were randomly assigned to a control group (CON), a weight gain prevention intervention delivered by a registered dietitian (RDG), or a weight gain prevention intervention delivered by a counselor (CSG). The weight gain prevention interventions delivered to the RDG and CSG were identical in content and materials, but differed in the credentialing of the group leader. Women randomized to the CON received no intervention.

Full details of the intervention have been described elsewhere [40]. Women in the RDG and CSG attended a total of 24 nutrition education sessions over the course of the 1-year intervention period. All sessions were 1-h in length and emphasized portion control, planning ahead and vegetable consumption [42, 43]. For the first 16 weeks of the intervention (months 1–4), participants attended weekly sessions; for the remaining 8 months of the study (months 5–12), participants attended monthly sessions [44]. Weekly sessions focused on general nutrition education topics, including basic nutrition and food groups, food selection and preparation, recipe modification, nutritious snack choices and snacking and nutrient density, among others, while monthly sessions addressed other areas of lifestyle behavior such as stress management, problem solving and motivation [40, 42–47]. Six session times were offered each week/month per group. Participants were permitted to attend the session day and time that worked best for them during the respective week/month. Dates, times and building location were matched between RDG and CSG to ensure all participants had the same opportunities to attend sessions.

Education sessions were randomly selected for process evaluation using investigator-established criteria to assess fidelity; all selected sessions were evaluated by the same process observer. The number of participants attending each session was recorded, as was the start and end time. The fidelity checklist included educator-oriented items along with content-related items. The process observer rated the educator using a ‘yes/no’ rating system on items such as preparedness, familiarity, accuracy and ability to respond appropriately to questions. A comment box was also used to note general feedback on these items as deemed relevant by the process observer. Content-related items addressed whether underlying key concepts (portion control, vegetable consumption, planning ahead) and problem-solving were covered. The process observer also recorded comments regarding challenges or difficulties of the education sessions (i.e., technological problems, outside distractions). These evaluations assessed whether content for each session was presented and delivered appropriately and that all session activities were completed.

Four female registered dietitians with <5 years of experience delivered the intervention to RDG participants. Four female graduate teaching assistants from the University of Illinois at Urbana-Champaign in programs not related to nutrition and dietetics delivered the intervention to CSG participants. Counselors were trained by one registered dietitian, including a code of conduct, before the start of the intervention. At least one week in advance of each session, all leaders were provided with the PowerPoint slides and script that included directions for session activities. Counselors also met with the registered dietitian to receive a brief overview of session handouts and activities and to have any questions answered.

To eliminate the potential for leader bias or participant attachment to a particular dietitian or counselor, education leaders rotated through class days and times to ensure equal interactions between participants in each group and each of the four respective group leaders. Compliance with the intervention was defined as attending >85 % of education sessions. For women who could not attend an education session, a virtual make-up session (PowerPoint slides and supporting materials), accompanied by a short quiz, was offered. Women were deemed compliant if the quiz was accurately completed and returned to the primary investigator. Women were not informed of the credentials of session leaders until completion of the 1-year intervention.

### Testing sessions

During the 1-year intervention, women participated in testing sessions (between 7:00 and 9:30 AM) before the intervention (baseline) and at month 3, month 6, month 9 and month 12. Anthropometric measurements, blood pressure, dietary intake, physical activity and biochemical markers of health were collected at each measurement interval. Upon completion of each testing session, women received a $10 gift card.

### Anthropometrics

A calibrated scale-mounted stadiometer (Seca 700, Hanover, MD, USA) was used to measure standing height (cm). Body weight (BW;kg), fat mass (FM;kg) and body fat percentage (BF%) were measured using a calibrated scale (Tanita 410GS, Arlington Heights, IL, USA). Height and BW measurements were used to calculate BMI (kg/m^2^) for each participant. Two measurements each of waist circumference (cm) and hip circumference (cm) were taken to the nearest 0.1 cm using a retractable measuring tape (Gulik II, Country Technology, Inc, Gay Mills, WI) and averaged. Waist circumference was measured at the narrowest point of the waist, approximately one inch above the navel, and hip circumference was measured at the widest part of the buttocks [43]. Waist and hip circumference measurements were used to calculate waist-to-hip ratio. One research team member conducted all height and BW measurements at all time intervals, and a second research team member performed all waist and hip circumference measurements at all time intervals.

### Blood pressure

A trained study investigator measured seated systolic and diastolic blood pressure (mm Hg) using a standard sphygmomanometer (Baumanometer®Desk Model, Copiague, NY, USA) at all testing sessions. Two blood pressure readings were taken with a 2–3 min rest period between readings; the average systolic arterial pressure and average diastolic arterial pressure were recorded. Resting heart rate was also measured by pulse palpitation following a 5-min rest period.

### Dietary intake

Four-day food records were used to estimate dietary intake. In the week before each testing session, participants recorded all foods and beverages, including portion sizes, consumed for three weekdays and one weekend day [40]. The Nutrition Data System for Research dietary analysis software (Nutrition Coordinating Center, Minneapolis, MN, USA) was used to analyze food records to estimate average daily intake of total energy (kcal/day), carbohydrate (g/day; %), protein (g/day; %), fat (g/day; %), fiber (g/day) and food groups (svgs/day).

### Physical activity

The Stanford 7-Day Physical Activity Recall Scale [48] was used to estimate physical activity. For seven consecutive days before each testing sessions, participants recorded the number of hours spent engaged in moderate, hard, and very hard activities, screen time (television, computer) and hours slept [48]. Minutes of physical activity per day were estimated by summing total minutes of moderate, hard, and very hard activity and dividing by seven. Calories expended per day were estimated by converting activities into metabolic equivalents (METs; hr/d) [40, 43].

### Biochemical markers of health

Venous blood samples were collected from each participant following a 12-h fast by a trained phlebotomist. Whole blood sat at room temperature for ≤60 min, after which samples were centrifuged at 1252 × *g* for 10 min at room temperature. Serum was stored at −80^0^C until completion of bioassays for glucose, insulin, low-density lipoprotein cholesterol (LDL-C), high-density lipoprotein cholesterol (HDL-C), total cholesterol and triglycerides (TG).

Spectrophotometry was used to measure serum glucose (mg/dL), total cholesterol (mg/dL), HDL-C (mg/dL) and TG (mg/dL) concentrations (all Stanbio Labs Boerne, TX, USA). Total cholesterol, HDL-C and TG concentrations were used to calculate LDL-C concentration (mg/dL) using the equation: LDL-C = total cholesterol - HDL-C - (TG/5) [49]. Enzyme-linked immunosorbent assay was used to measure serum insulin (μU/mL; LINCO Research, St Charles, MO, USA). All serum samples were analyzed in duplicate for each study interval. Intra-assay coefficients of variations for serum glucose, total cholesterol, HDL-C and TG were 5.2, 5.4, 4.9 and 7.2 %, respectively.

### Statistical analyses

A 3x5 (3 treatment groups x 5 time intervals) analysis of covariance (ANCOVA) with repeated measures on the time factor was used to assess differences in outcome measures between and within treatment groups. Baseline age, BW and BMI were different between the three groups (RDG, CSG and CON) and entered as covariates in all analyses examining effects of intervention. If sphericity was violated, the Greenhouse-Geisser correction was used. The interaction of group (treatment) x interval (time) was assessed if a main effect of group or time was detected. Bonferroni adjustments for multiple comparisons were completed when significant group, time or group x time interactions were found. All participants completing baseline testing (*n* = 81) were included in the intention-to-treat analyses, and the last observation carried forward approach was employed. A secondary efficacy analysis of only study completers also was conducted (*n* = 48).

Before conducting data analyses, outliers were identified for each outcome variable using the outlier labeling rule [50, 51] and excluded from all analyses specific to that variable. All data analyses were conducted using the Statistical Package for the Social Sciences (version 22.0, 2013, IBM Corp, Armonk, NY, USA). Statistical tests were two-sided, and significance was set at *P* < 0.01.

## Results

Of the 330 women that responded to recruitment efforts between June-August 2014, 266 women met prescreening criteria and were sent additional screening materials and informed consent, which were returned by 146 women [40]. After review of materials by investigators, 102 women met eligibility criteria, and 97 of these women attended an informational session.

**Fig. 1:**
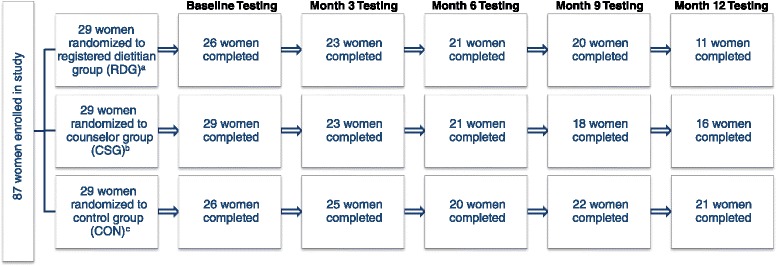
Participant flow diagram through a 1-year randomized controlled trial of weight gain prevention using registered dietitian-led (RDG) or counselor-led (CSG) nutrition education or control (CON) groups. ^a^Reasons for withdrawal in RDG: family emergency (*n*=1 before baseline testing, *n*=1 before month 3 testing, *n*=2 before month 12 testing), lost to follow up (*n*=2 before baseline testing, *n*=1 before month 6 testing, *n*=1 before month 12 testing), moved out of the area (*n*=6 before month 12 testing), pregnancy (*n*=1 before month 9 testing, *n*=1 before month 12 testing), time (*n*=2 before month 3 testing); 1 participant in the RDG did not attend the month 6 testing session due to a family emergency, but completed all remaining testing sessions; 1 participant in the RDG did not attend the month 9 testing session due to a family emergency, but completed all remaining testing sessions. ^b^Reasons for withdrawal in CSG: lost to follow up (*n*=1 before month 6 testing, *n*=1 before month 12 testing), moved out of the area (*n*=1 before month 9 testing, *n*=2 before month 12 testing), personal health issues (*n*=1 before month 9 testing), time (*n*=4 before month 3 testing), uncomfortable with study (*n*=1 before month 3 testing), undisclosed (*n*=1 before month 3 testing), Zung >50 (*n*=1 before month 12 testing). ^c^Reasons for withdrawal in CON: family issues (*n*=1 before month 12 testing), moved out of the area (*n*=1 before month 12 testing), pregnancy (*n*=1 before month 6 testing), time (*n*=3 before baseline testing, *n*=1 before month 3 testing), Zung >50 (*n*=1 before month 6 testing); 3 participants in the CON did not attend month 9 testing (illness *n*=2, time *n*=1), but returned for month 12 testing

Figure 1 displays the flow diagram of enrollment and study completion of women in the current randomized controlled trial of weight gain prevention. Eighty-seven women were enrolled and randomized to the RDG (*n* = 29), CSG (*n* = 29) or CON (*n* = 29) group. Baseline testing was completed by 81 healthy premenopausal women (White, non-Hispanic, *n* = 53; Black, non-Hispanic, *n* = 10; Asian, *n* = 8; Non-white Hispanic or Latino, *n* = 4; Other, including multiracial, *n* = 6). Seventy-one women completed month 3 testing, 62 women completed month 6 testing, 60 women completed month 9 testing, and 48 women completed month 12 testing. The 81 women completing baseline testing (mean ± SD, age: 31.4 ± 8.1 y; BW: 76.1 ± 19.0 kg; BMI: 27.9 ± 6.8 kg/m^2^) were included in intention-to-treat analyses. Women who completed the intervention (*n* = 48; mean ± SD, age: 33.4 ± 7.2 y; BW: 79.7 ± 19.7 kg; BMI: 29.5 ± 7.2 kg/m^2^) were significantly older (*P* < 0.01) as compared to women who did not complete the intervention (*n* = 33; mean ± SD, age: 28.4 ± 8.6 y; BW: 70.7 ± 17.0 kg; BMI: 25.6 ± 5.6 kg/m^2^); study completers also were heavier as compared to women who did not complete the study, but this was not significant (*P* > 0.01). When controlling for baseline age, BW and BMI, group assignment did not have an effect on participant dropout (*P* > 0.01).

### Weight gain prevention

Approximately 62.0 % of the original sample (*n* = 50) and 56.3 % of completers (*n* = 27) maintained BW (±3 %) over the intervention period. Using intention-to-treat analysis, 65.0 % of the RDG (*n* = 17), 65.5 % of the CSG (*n* = 19) and 53.8 % of the CON (*n* = 14) group met weight gain prevention criteria and were classified as successful. This distribution was similar among completers with 63.6 % of the RDG (*n* = 7), 56.3 % of the CSG (*n* = 9) and 52.4 % of the CON (*n* = 11) group meeting weight gain prevention criteria (*P* > 0.01).

### Process observation and education session compliance

Twenty-two RDG and 17 CSG education sessions were observed. On average (±SD), sessions lasted 39.1 (±11.1) min; however, RDG sessions were significantly longer than CSG sessions [43.3 (±9.2) vs. 33.5 (±11.2) min, *P* < 0.01]. On average (±SD), 2.9 (±1.6) participants attended each session, and this did not differ between groups. Based on the fidelity checklist, participants in both groups were equally engaged in education sessions, and vegetable consumption, portion control and planning ahead for food intake were addressed equally across all sessions. The process observer perceived that the registered dietitians were more likely to give specific scenarios and reinforce points made in various education sessions than counselors. Additionally, the process observer perceived that counselors tended to read from the script, while registered dietitians were more familiar with the topics and able to discuss without relying on supporting materials.

Weekly, monthly and overall compliance did not differ by group assignment. Compliance was defined as attendance at >85 % of education sessions (14/16 for weekly sessions, 7/8 for monthly sessions, 21/24 overall). Using this criteria, 88.6 % (*n* = 39) of women randomized to the RDG and CSG who were still enrolled after 16 weeks (*n* = 44) were deemed compliant with weekly attendance (RDG = 20; CSG = 19), and 81.5 % (*n* = 22) of the RDG and CSG who completed the intervention (*n* = 27) were compliant with monthly attendance (RDG = 10; CSG = 12). Overall, 88.5 % (*n* = 23) of women who were randomized to the RDG and CSG and completed month 12 testing (*n* = 27) were compliant (RDG = 10; CSG = 13).

### Anthropometric measurements

There were no significant group differences for BW, BMI, FM, waist circumference, hip circumference or waist-to-hip ratio (Table 1). BF% was significantly lower in the RDG compared to the CSG and CON groups at all intervals. Women in all groups were able to prevent gains in BW, BMI, FM, BF%, waist circumference, hip circumference and waist-to-hip ratio over time, and there was no time effect for these anthropometric measurements. Results were similar with efficacy analysis (data not shown); however, the P-value for group differences in BF% increased to *P* < 0.01 (*P* < 0.001 for intention-to-treat analysis).

**Table 1:** Anthropometric measurements of premenopausal women in a 1-year randomized controlled trial of weight gain prevention in which women were randomized to a registered dietitian-led nutrition education group (RDG), a counselor-led nutrition education group (CSG) or a control group (CON)

		Baseline	Month 3	Month 6	Month 9	Month 12	
Variable	Group						*P*-value
Body weight (kg)	RDG (*n* = 26)	73.9 ± 1.6	74.7 ± 1.7	74.9 ± 1.8	74.7 ± 1.9	75.2 ± 1.9	Group = NS
	CSG (*n* = 29)	74.2 ± 1.1	74.4 ± 1.1	75.0 ± 1.2	74.7 ± 1.3	75.1 ± 1.3	Time = NS
	CON (*n* = 25)	77.9 ± 1.9	76.8 ± 2.0	76.6 ± 2.1	77.5 ± 2.2	77.2 ± 2.2	G x T = NS
Body mass index (kg/m^2^)	RDG (*n* = 26)	26.1 ± 0.5	26.2 ± 0.6	26.4 ± 0.6	26.5 ± 0.6	26.6 ± 0.6	Group = NS
	CSG (*n* = 29)	27.4 ± 0.4	27.5 ± 0.4	27.8 ± 0.4	27.7 ± 0.4	27.8 ± 0.5	Time = NS
	CON (*n* = 25)	29.3 ± 0.7	29.1 ± 0.7	28.9 ± 0.7	29.1 ± 0.7	29.1 ± 0.8	G x T = NS
Fat mass (kg)	RDG (*n* = 26)	26.6 ± 0.5	27.4 ± 0.6	27.9 ± 0.8	28.1 ± 0.8	27.7 ± 0.9	Group = NS
	CSG (*n* = 29)	27.0 ± 0.3	27.8 ± 0.4	28.3 ± 0.6	28.0 ± 0.6	28.2 ± 0.6	Time = NS
	CON (*n* = 25)	28.4 ± 0.5	28.4 ± 0.7	28.0 ± 1.0	28.3 ± 1.0	28.6 ± 1.1	G x T = NS
Body fat percentage (%)	RDG (*n* = 26)	31.2 ± 0.8	32.1 ± 0.9	32.6 ± 1.0	33.1 ± 1.0	32.1 ± 1.0	Group = <0.001
	CSG (*n* = 29)	35.1 ± 0.6^a^	36.0 ± 0.6^a^	36.4 ± 0.7^a^	36.0 ± 0.7^a^	36.0 ± 0.6^a^	Time = NS
	CON (*n* = 25)	36.9 ± 1.0^a^	37.3 ± 1.0^a^	36.7 ± 1.2^a^	36.8 ± 1.1^a^	38.0 ± 1.1^a^	G x T = NS
Waist circumference (cm)	RDG (*n* = 26)	80.2 ± 1.3	80.4 ± 1.2	80.3 ± 1.3	80.2 ± 1.4	81.5 ± 1.3	Group = NS
	CSG (*n* = 29)	82.5 ± 0.9	82.3 ± 0.8	82.7 ± 0.9	82.4 ± 0.9	82.8 ± 0.9	Time = NS
	CON (*n* = 25)	85.9 ± 1.5	84.3 ± 1.4	83.6 ± 1.5	83.6 ± 1.6	83.7 ± 1.5	G x T = NS
Hip circumference (cm)	RDG (*n* = 26)	107.0 ± 1.3	108.9 ± 1.2	108.9 ± 1.2	107.2 ± 1.3	107.3 ± 1.4	Group = NS
	CSG (*n* = 29)	110.1 ± 0.9	110.3 ± 0.8	110.1 ± 0.8	109.3 ± .08	109.5 ± .09	Time = NS
	CON (*n* = 24)	111.8 ± 1.5	111.5 ± 1.4	111.9 ± 1.4	111.4 ± 1.5	112.0 ± 1.7	G x T = NS
Waist-to-hip ratio	RDG (*n* = 26)	0.74 ± 0.02	0.73 ± 0.02	0.72 ± 0.01	0.73 ± 0.02	0.74 ± 0.01	Group = NS
	CSG (*n* = 29)	0.75 ± 0.01	0.74 ± 0.01	0.75 ± 0.01	0.75 ± 0.01	0.75 ± 0.01	Time = NS
	CON (*n* = 25)	0.78 ± 0.02	0.76 ± 0.02	0.75 ± 0.02	0.76 ± 0.02	0.76 ± 0.02	G x T = NS

*P*-values using analysis of covariance, adjusted for baseline age, body weight and body mass index, with repeated measures on the time factor, using *P* < 0.01 for statistical significance; data presented as adjusted means ± standard error of the mean

^a^different from RDG

G x T = group x time interaction; NS = not significant

### Blood pressure

Group assignment had no significant effect on resting heart rate, systolic blood pressure or diastolic blood pressure (Table 2). Significant changes in systolic blood pressure were observed over time within the CON group from month 6 to 9 (↑2.4 %) and month 6 to 12 (↓2.7 %); these changes were significantly different from the RDG (↓5.6 %, 6 to 9 month; ↓1.2 %, 6 to 12 month) and CSG (↓2.3 %, 6 to 9 month; ↓2.0 %, 6 to 12 month) groups {group x time interaction [F(7.1, 264.5) = 2.7, *P* < 0.01]}. The efficacy analysis revealed a significant increase in systolic blood pressure (*P* < 0.01) for the CON group from month 6 to 9 (data not shown). The group x time interaction for systolic blood pressure was no longer significant in the efficacy analysis.

**Table 2:** Resting heart rate and blood pressure measurements of premenopausal women in a 1-year randomized controlled trial of weight gain prevention in which women were randomized to a registered dietitian-led nutrition education group (RDG), a counselor-led nutrition education group (CSG) or a control group (CON)

		Baseline	Month 3	Month 6	Month 9	Month 12	
Variable	Group						*P*-value
Resting heart rate (bpm)	RDG (*n* = 23)	64.2 ± 0.74	73.0 ± 2.8	67.5 ± 2.0	64.5 ± 2.2	64.1 ± 1.6	Group = NS
	CSG (*n* = 22)	65.1 ± 0.52	65.1 ± 1.9	62.6 ± 1.4	63.1 ± 1.6	61.9 ± 1.2	Time = NS
	CON (*n* = 20)	65.2 ± 0.89	58.7 ± 3.3	64.7 ± 2.4	65.7 ± 2.7	58.8 ± 2.0	G x T = NS
Resting systolic blood pressure (mm Hg)	RDG (*n* = 26)	107.2 ± 2.8	103.3 ± 2.7	103.9 ± 2.4	98.1 ± 2.6	102.7 ± 2.5	Group = NS
	CSG (*n* = 29)	109.0 ± 1.9	106.8 ± 1.8	108.0 ± 1.6	105.5 ± 1.8	105.8 ± 1.7	Time = <0.001
	CON (*n* = 25)	102.1 ± 3.3	106.2 ± 3.2	107.7 ± 2.9	110.3 ± 3.1^a^	104.9 ± 3.0^a^	G x T = <0.01
Resting diastolic blood pressure (mm Hg)	RDG (*n* = 26)	68.9 ± 2.5	66.1 ± 2.3	64.7 ± 2.3	63.9 ± 2.1	63.5 ± 2.2	Group = NS
	CSG (*n* = 29)	72.7 ± 1.7	69.2 ± 1.5	68.1 ± 1.6	67.3 ± 1.4	66.9 ± 1.5	Time = NS
	CON (*n* = 25)	70.9 ± 2.9	69.2 ± 2.7	62.4 ± 2.7	65.5 ± 2.5	67.3 ± 2.5	G x T = NS

*P*-values using analysis of covariance, adjusted for baseline age, body weight and body mass index, with repeated measures on the time factor, using *P* < 0.01 for statistical significance; data presented as adjusted means ± standard error of the mean

^a^different from Month 6

G x T = group x time interaction; NS = not significant

### Dietary intake

Dietary intake data are displayed in Table 3. Estimated carbohydrate intake (%) was 7.7 % more in the RDG vs. CON group at month 9, and estimated fat intake (%) was significantly less in the RDG vs. CON group and CSG vs. CON group at month 3 (by 10.0 %) and 9 (by 8.1 %), respectively. Estimated protein intake (%) did not differ between groups. Estimated total energy intake, total and % carbohydrate, total and % protein, total and % fat and total fiber did not change significantly over time.

**Table 3:** Estimated macronutrient and food group intakes and estimated energy expenditure of premenopausal women in a 1-year randomized controlled trial of weight gain prevention in which women were randomized to a registered dietitian-led nutrition education group (RDG), a counselor-led nutrition education group (CSG) or a control group (CON)

		Baseline	Month 3	Month 6	Month 9	Month 12	
Variable	Group						*P*-value
Energy intake (kcal/d)	RDG (*n* = 26)	1783 ± 67	1723 ± 59	1675 ± 62	1703 ± 63	1599 ± 63	Group = NS
	CSG (*n* = 29)	1796 ± 63	1703 ± 56	1804 ± 58	1742 ± 60	1843 ± 60	Time = NS
	CON (*n* = 26)	1806 ± 67	1627 ± 59	1666 ± 62	1675 ± 63	1712 ± 63	G x T = NS
Total carbohydrate (g/d)	RDG (*n* = 26)	219 ± 9	205 ± 8	202 ± 8	208 ± 8	186 ± 8	Group = NS
	CSG (*n* = 29)	214 ± 9	207 ± 7	217 ± 8	208 ± 8	219 ± 8	Time = NS
	CON (*n* = 26)	205 ± 9	183 ± 8	180 ± 8	184 ± 8	194 ± 8	G x T = NS
Total protein (g/d)	RDG (*n* = 26)	71.2 ± 3.2	76.0 ± 2.9	71.8 ± 3.0	69.4 ± 3.0	68.6 ± 3.0	Group = NS
	CSG (*n* = 29)	70.0 ± 3.0	69.2 ± 2.7	72.8 ± 2.8	71.7 ± 2.8	75.1 ± 2.8	Time = NS
	CON (*n* = 26)	74.6 ± 3.2	68.5 ± 2.9	71.0 ± 3.0	73.6 ± 3.0	72.2 ± 3.0	G x T = NS
Total fat (g/d)	RDG (*n* = 26)	70.8 ± 3.6	68.4 ± 3.3	66.2 ± 3.3	68.2 ± 3.2	65.7 ± 3.3	Group = NS
	CSG (*n* = 29)	73.3 ± 3.4	67.4 ± 3.1	71.8 ± 3.1	68.3 ± 3.0	72.0 ± 3.1	Time = NS
	CON (*n* = 26)	76.5 ± 3.6	70.7 ± 3.3	70.1 ± 3.3	72.9 ± 3.2	72.7 ± 3.3	G x T = NS
Total fiber (g/d)	RDG (*n* = 26)	18.9 ± 1.0	20.7 ± 0.9	19.6 ± 1.0	19.6 ± 1.0	16.7 ± 0.9	Group = NS
	CSG (*n* = 29)	19.3 ± 1.0	17.6 ± 0.8	19.9 ± 0.9	19.2 ± 0.9	18.5 ± 0.8	Time = NS
	CON (*n* = 26)	17.5 ± 1.0	17.1 ± 0.9	18.4 ± 1.0	17.2 ± 1.0	17.9 ± 0.9	G x T = NS
Percentage carbohydrate (%kcal/d)	RDG (*n* = 26)	48.2 ± 1.1	47.0 ± 1.1	47.5 ± 1.0	47.8 ± 1.1	46.1 ± 1.0	Group = <0.01
	CSG (*n* = 29)	47.3 ± 1.1	47.7 ± 1.0	46.9 ± 1.0	46.5 ± 1.0	46.2 ± 1.0	Time = NS
	CON (*n* = 26)	45.4 ± 1.1	44.1 ± 1.1	44.1 ± 1.0	43.3 ± 1.1^a^	44.5 ± 1.0	G x T = NS
Percentage protein (%kcal/d)	RDG (*n* = 26)	16.5 ± 0.6	18.0 ± 0.6	17.6 ± 0.6	16.7 ± 0.6	17.6 ± 0.6	Group = NS
	CSG (*n* = 29)	15.8 ± 0.5	16.7 ± 0.5	16.7 ± 0.5	17.0 ± 0.5	16.8 ± 0.5	Time = NS
	CON (*n* = 26)	16.6 ± 0.6	17.4 ± 0.6	17.7 ± 0.6	18.1 ± 0.6	17.2 ± 0.6	G x T = NS
Percentage fat (%kcal/d)	RDG (*n* = 26)	33.8 ± 0.9	34.0 ± 0.9	33.8 ± 0.9	34.6 ± 0.9	35.1 ± 0.9	Group = <0.01
	CSG (*n* = 29)	35.1 ± 0.9	34.5 ± 0.9	34.9 ± 0.8	34.7 ± 0.8	34.6 ± 0.8	Time = NS
	CON (*n* = 26)	36.5 ± 0.9	37.7 ± 0.9^a^ ^b^	36.8 ± 0.9	37.7 ± 0.9^a^ ^b^	36.9 ± 0.9	G x T = NS
Fruit (svgs/d)	RDG (*n* = 26)	1.9 ± 0.21	1.7 ± 0.15	2.1 ± 0.18	1.9 ± 0.17	2.0 ± 0.22	Group = <0.01
	CSG (*n* = 29)	1.4 ± 0.20	1.2 ± 0.14	1.4 ± 0.17	1.3 ± 0.16	1.5 ± 0.20	Time = NS
	CON (*n* = 26)	1.6 ± 0.21	1.0 ± 0.15^a^	1.1 ± 0.18^a^	1.0 ± 0.17^a^	1.3 ± 0.22^a^	G x T = NS
Vegetables (svgs/d)	RDG (*n* = 26)	3.4 ± 0.25	3.2 ± 0.21	3.3 ± 0.24	3.0 ± 0.22	3.3 ± 0.25	Group = NS
	CSG (*n* = 29)	3.3 ± 0.24	2.9 ± 0.20	2.2 ± 2.23	3.2 ± 0.21	3.1 ± 0.23	Time = NS
	CON (*n* = 26)	3.3 ± 0.23	3.3 ± 0.21	3.3 ± 0.24	3.3 ± 0.22	3.4 ± 0.24	G x T = NS
Whole grains (svgs/d)	RDG (*n* = 26)	1.7 ± 0.22	1.7 ± 0.19	1.6 ± 0.18	1.4 ± 0.18	1.4 ± 0.18	Group = NS
	CSG (*n* = 29)	1.8 ± 0.21	1.7 ± 0.18	1.7 ± 0.17	1.8 ± 0.17	1.6 ± 0.17	Time = NS
	CON (*n* = 26)	1.8 ± 0.23	1.7 ± 0.19	1.7 ± 0.18	1.6 ± 0.18	1.8 ± 0.18	G x T = NS
Refined grains (svgs/d)	RDG (*n* = 26)	3.9 ± 0.32	3.6 ± 0.29	3.6 ± 0.30	4.0 ± 0.32	3.4 ± 0.31	Group = NS
	CSG (*n* = 29)	4.4 ± 0.30	4.3 ± 0.27	4.3 ± 0.28	4.1 ± 0.30	4.6 ± 0.30	Time = NS
	CON (*n* = 26)	4.2 ± 0.32	3.4 ± 0.29	3.3 ± 0.30	3.6 ± 0.31	3.8 ± 0.31	G x T = NS
Lean meats (svgs/d)	RDG (*n* = 26)	1.7 ± 0.25	2.1 ± 0.25	2.1 ± 0.24	1.8 ± 0.25	2.1 ± 0.26	Group = NS
	CSG (*n* = 29)	1.4 ± 0.24	1.4 ± 0.23	1.7 ± 0.23	1.9 ± 0.23	2.0 ± 0.25	Time = NS
	CON (*n* = 26)	1.8 ± 0.25	1.7 ± 0.25	1.7 ± 0.24	2.0 ± 0.25	1.9 ± 0.26	G x T = NS
Non-meat protein sources (svgs/d)	RDG (*n* = 26)	1.7 ± 0.25	2.5 ± 0.22	2.4 ± 0.25	2.2 ± 0.24	1.8 ± 0.19	Group = <0.001
	CSG (*n* = 29)	1.5 ± 0.23	1.0 ± 0.21^a^	1.4 ± 0.24	1.4 ± 0.24	1.1 ± 0.18	Time = NS
	CON (*n* = 26)	1.3 ± 0.25	1.5 ± 0.22^a^	1.5 ± 0.25	1.4 ± 0.24	1.3 ± 0.19	G x T = NS
Low-fat and fat-free dairy (svgs/d)	RDG (*n* = 26)	0.55 ± 0.08	0.75 ± 0.10	0.80 ± 0.10	0.66 ± 0.09	0.71 ± 0.09	Group = NS
	CSG (*n* = 29)	0.78 ± 0.08	0.89 ± 0.10	0.83 ± 0.09	0.64 ± 0.09	0.86 ± 0.09	Time = NS
	CON (*n* = 26)	0.68 ± 0.08	0.79 ± 0.10	0.66 ± 0.10	0.84 ± 0.09	0.64 ± 0.09	G x T = NS
Energy expenditure (kcal/d)	RDG (*n* = 26)	1202 ± 126	1223 ± 113	1107 ± 122	984 ± 103	1038 ± 107	Group = NS
	CSG (*n* = 27)	1168 ± 85	1040 ± 76	1007 ± 82	1062 ± 69	1074 ± 72	Time = NS
	CON (*n* = 21)	1103 ± 163	1066 ± 146	1163 ± 158	1229 ± 133	1261 ± 139	G x T = NS

*P*-values using analysis of covariance, adjusted for baseline age, body weight and body mass index, with repeated measures on the time factor, using *P* < 0.01 for statistical significance; data presented as adjusted means ± standard error of the mean

^a^different from RDG; ^b^different from CSG

G x T = group x time interaction; NS = not significant

Estimated servings of fruits, vegetables, whole and refined grains, lean meats and non-meat protein sources and low-fat and fat-free dairy did not change over time. Fruit intake was significantly more in the RDG vs. CON group at months 3 (by 0.7 svgs/d), 6 (by 1.0 svgs/d), 9 (by 0.9 svgs/d) and 12 (by 0.7 svgs/d). Intake of non-meat protein sources was significantly less in the CSG (by 1.5 svgs/d) and CON (by 1.0 svgs/d) groups as compared to the RDG group at month 3. Group x time interactions were not found for any macronutrients or food group servings. The efficacy analysis revealed no significant differences in macronutrient or food group serving intakes between groups or over time (data not shown).

### Physical activity

Total energy expenditure did not significantly change over time and was not significantly different by group (Table 3). Results from the efficacy analysis (data not shown) were congruent with intention-to-treat findings.

### Biochemical markers of health

No significant group differences were observed for total cholesterol, HDL-C, LDL-C, TG, glucose or insulin concentrations, and there were no significant changes for any of these biomarkers over time (Table 4). Similar results were found with the efficacy analysis (data not shown).

**Table 4:** Blood lipid, glucose and insulin concentrations in premenopausal women in a 1-year randomized controlled trial of weight gain prevention in which women were randomized to a registered dietitian-led nutrition education group (RDG), a counselor-led nutrition education group (CSG) or a control group (CON)

		Baseline	Month 3	Month 6	Month 9	Month 12	
Variable	Group						*P*-value
Total cholesterol (mg/dL)	RDG (*n* = 26)	169.0 ± 8.8	177.0 ± 9.5	178.7 ± 8.7	181.7 ± 8.8	175.8 ± 8.5	Group = NS
	CSG (*n* = 28)	163.0 ± 6.0	176.8 ± 6.5	174.3 ± 5.9	178.0 ± 6.0	176.0 ± 5.8	Time = NS
	CON (*n* = 24)	156.2 ± 10.5	166.6 ± 11.3	170.4 ± 10.4	173.8 ± 10.6	160.7 ± 10.2	G x T = NS
High-density lipoprotein cholesterol (mg/dL)	RDG (*n* = 26)	54.6 ± 3.7	54.1 ± 3.6	51.4 ± 3.4	53.1 ± 3.6	49.8 ± 3.7	Group = NS
	CSG (*n* = 29)	52.5 ± 2.5	51.7 ± 2.4	51.6 ± 2.2	51.8 ± 2.4	50.4 ± 2.4	Time = NS
	CON (*n* = 24)	45.6 ± 4.4	45.8 ± 4.3	47.8 ± 4.0	48.9 ± 4.2	50.5 ± 4.4	G x T = NS
Low-density lipoprotein cholesterol (mg/dL)	RDG (*n* = 26)	97.2 ± 7.0	104.1 ± 7.3	109.7 ± 7.1	111.5 ± 7.6	106.3 ± 6.8	Group = NS
	CSG (*n* = 28)	96.9 ± 4.7	110.8 ± 5.0	107.8 ± 4.8	111.9 ± 5.1	111.5 ± 4.6	Time = NS
	CON (*n* = 23)	95.3 ± 8.5	111.9 ± 9.0	113.5 ± 8.6	111.8 ± 9.3	99.0 ± 8.3	G x T = NS
Triglycerides (mg/dL)	RDG (*n* = 25)	54.2 ± 8.8	54.4 ± 7.7	56.4 ± 7.4	55.4 ± 8.7	62.2 ± 8.3	Group = NS
	CSG (*n* = 28)	64.7 ± 5.8	63.4 ± 5.1	64.3 ± 4.9	68.1 ± 5.7	67.3 ± 5.5	Time = NS
	CON (*n* = 22)	86.0 ± 10.8	65.4 ± 9.4	62.9 ± 9.1	79.5 ± 10.6	73.0 ± 10.2	G x T = NS
Glucose (mg/dL)	RDG (*n* = 23)	82.1 ± 4.7	84.6 ± 6.5	93.3 ± 2.3	96.7 ± 3.0	91.5 ± 2.8	Group = NS
	CSG (*n* = 25)	87.4 ± 3.1	86.1 ± 4.4	92.2 ± 1.5	92.5 ± 2.0	91.8 ± 1.9	Time = NS
	CON (*n* = 23)	90.6 ± 5.3	80.0 ± 7.4	90.5 ± 2.6	88.2 ± 3.4	89.4 ± 3.1	G x T = NS
Insulin (μU/mL)	RDG (*n* = 25)	4.5 ± 0.59	6.1 ± 0.82	4.9 ± 0.67	5.0 ± 0.57	3.0 ± 0.67	Group = NS
	CSG (*n* = 28)	5.1 ± 0.40	5.9 ± 0.56	5.2 ± 0.46	5.3 ± 0.39	5.0 ± 0.45	Time = NS
	CON (*n* = 21)	5.2 ± 0.74	5.2 ± 1.00	5.6 ± 0.84	5.3 ± 0.71	8.0 ± 0.83	G x T = NS

Fasting serum used to measure all biomarker concentrations

*P*-values using analysis of covariance, adjusted for baseline age, body weight and body mass index, with repeated measures on the time factor, using *P* < 0.01 for statistical significance; data presented as adjusted means ± standard error of the mean

G x T = group x time interaction; NS = not significant

## Discussion

This randomized controlled trial aimed to prevent weight gain in healthy premenopausal women over one year and to compare the effects of an intervention delivered by a registered dietitian to a counselor on weight gain prevention in the same sample of women. Although women randomized to a nutrition education intervention were able to maintain BW within ±3 %, women who did not receive a nutrition education intervention also were able to maintain BW within ±3 %. Despite neither hypothesis being supported, approximately 62 % of women enrolled in the study were able to maintain BW over the 1-year intervention. Though a greater percentage of women in the RDG group (65 %; mean ± SD absolute BW change: 1.4 ± 2.9 kg; mean ± SD relative BW change: 0.79 ± 1.6 %) and CSG (66 %; mean ± SD absolute BW change: 0.50 ± 3.2 kg; mean ± SD relative BW change: 0.41 ± 2.3 %) were successful in weight gain prevention as compared to the CON group (54 %; mean ± SD absolute BW change: 0.71 ± 3.8 kg; mean ± SD relative BW change: 0.61 ± 2.6 %), these group differences were not significant. The mean ± SD absolute and relative changes in BW for the entire sample were 0.85 ± 3.3 kg and 0.61 ± 2.6 %, respectively. For the typical participant that met weight gain prevention criteria, mean ± SD absolute BW change was −0.05 ± 1.14 kg and ranged from a relative BW change of −2.7 to 2.7 %. The lack of differences between the three groups demonstrates that women can, in fact, successfully prevent weight gain over one year. However, a large proportion of the population still struggles with weight gain, and further evaluation is needed to have a significant impact on the current obesity epidemic in the United States.

Overall findings from this study related to changes in BW and weight gain prevention are consistent with those of Pound of Prevention [28, 29], Levine and colleagues [30] and the Groningen Overweight and Lifestyle (GOAL) Study [31–33], which found interventions or treatments to have no significant effect on BW over time. In Pound of Prevention, a no-contact control group was compared to a group that received monthly nutrition education via newsletters and a group that received the same nutrition education plus lottery incentives for participation [28, 29]. Over a 3-year period, weight gain (~0.5 kg/y) did not differ significantly between groups [29], and mean BW gain after one year in women only (~0.7 kg/y) in Pound of Prevention [28] was similar to that of the current study (~0.5 kg/y). Similarly, no effect on BW (~0.8 kg/y increase) in normal weight and overweight women was observed by Levine and colleagues [30], who compared 15 group education sessions, 15 correspondence education lessons and an information-only control over three years. The GOAL Study randomized overweight and obese men and women with hypertension and/or dyslipidemia to receive usual care from a general practitioner or computer-guided lifestyle counseling from a nurse practitioner [31–33]. After one year, men randomized to the nurse practitioner group had a significantly greater weight loss (~–2.0 kg/y) as compared to the usual care group (~–0.1 kg/y) [31], but these differences were not apparent after three years (~–0.7 kg/y) [33]. In women, there was no difference between the two groups at either one year (~–1.4 kg/y) [31, 32] or three years (~–0.8 kg/y) [33].

When examining the prevalence of weight gain prevention or weight maintenance in the context of a population, it becomes somewhat problematic, as the definition of weight maintenance has not been consistently used. Jeffery and French found that 37 % of participants in Pound of Prevention maintained or lost weight, but investigators did not specify how “maintained” was defined [29]. A similar prevalence of weight gain prevention (40 %) was found by Levine and colleagues [30], who defined maintenance as at or below ±2 lb of baseline weight. The GOAL Study found a prevalence of 71.4 and 62.7 % at one [31] and three years [33], respectively, using a definition of <1 % BW gain [31–33]. The 62 % of women that were classified as successful at weight gain prevention in the current study may be slightly underestimated as the definition used in the current study is more conservative. Women were *only* classified as weight maintainers if percent BW change was ±3 %; therefore, women who lost >3 % of BW (i.e., “weight losers”) were classified as non-weight maintainers.

Only two of the five studies with published results that have examined weight gain prevention have been successful in preventing weight gain over time [27, 34]; final results of the Study of Novel Approaches to Weight Gain Prevention are not yet available [35]. Age has been identified as a predictor of weight gain prevention [27, 30] which may explain why a low-intensity nutrition education intervention via monthly newsletters was effective in producing a significantly greater BW change over one year when compared to a no-contact control in which the mean age of participants was 45.9 years [27]. The Shape Program [34] compared usual care to a primary care-based medium-intensity behavioral weight gain prevention intervention in premenopausal overweight and obese class 1 black women. Weight change was significantly larger in the intervention group compared to usual care at one year and 18 months; however, there were no differences between groups in any other outcome measures [34]. While findings of the Shape Program were significant, results are limited in generalizability due to race and socioeconomic status of the sample population.

The current randomized controlled trial of weight gain prevention builds upon the strengths and recommendations of previous weight gain prevention trials, but was unique in that it included only women and did not exclude on the basis of BMI (except for underweight). Treatment had a greater effect on weight gain prevention after one year in men in A Pound of Prevention [27] and the GOAL Study [31–33] which suggests women may need different types or intensities of interventions. Both of these interventions [27, 31–33] were relatively low in intensity, while the current trial was moderate in intensity. The nutrition education component of the current intervention included group education classes, as previous research has demonstrated that group therapy results in greater weight loss when compared to individual therapy, even among individuals who prefer individual counseling [52]. Jeffery and French [29] previously recommended that more attention should be given to frequency of messages, interactive components and motivational concerns, all of which were addressed in the current randomized controlled trial of weight gain prevention in women.

With further regard to the nutrition education component of the intervention, this is the first trial to test a weight gain prevention nutrition education intervention delivered by a registered dietitian compared to an individual without formal nutrition training. The absence of differences in main outcomes between the RDG and CSG suggests that the two interventions were equally effective in promoting weight gain prevention in this sample of healthy premenopausal women. Similar findings related to the use of peer or lay educators have been observed in programs and interventions aimed at reducing chronic disease risk factors [53–57]. Although it was hypothesized that the RDG would have less weight gain over time due to the specialized training of registered dietitians, the counselors were trained by a registered dietitian and received all lesson slides and materials, including scripts. These findings are similar to those of Katula and colleagues in which community health workers trained and supported by registered dietitians delivered a 24-month lifestyle intervention that resulted in significant reductions in BW, BMI, waist circumference, glucose, insulin and insulin resistance in individuals with pre-diabetes [55]. When provided with adequate training by registered dietitians, lay educators may be able to promote outcomes equal to those of registered dietitians. These findings present an opportunity for registered dietitians to expand their sphere of influence by training and supporting lay educators to have an impact on health promotion by providing accurate and credible information. By training these individuals, registered dietitians can help ensure that accurate information is communicated with the public.

As a focus on weight and weight loss has not produced positive long-term results in reducing the obesity epidemic, a continued focus on weight as the primary indicator of health may cause more harm than good by increasing preoccupation with food, increasing the likelihood of weight cycling and decreasing body image and self-esteem [2]. Therefore, collecting and examining other indicators of health, beyond BW and anthropometrics, is warranted. Weight is one component of metabolic health [58, 59] and other indicators of metabolic health should be considered when assessing an overall health status. Even in this sample of women who were overweight, on average, blood pressure, lipid levels and glucose were within normal ranges. Further, lifestyle and behavior changes can positively improve clinical health indicators, even in the absence of weight change [20–26]. Using only BW or BMI as proxies for health may misidentify healthy overweight and obese individuals as unhealthy and in need of treatment and unhealthy normal weight individuals as healthy and not in need of treatment [2, 58, 59]. Both of these situations may translate into increased healthcare costs through unnecessary treatment or worsening of conditions that were not identified due to the use of BW or BMI as the primary indicators of health. The current trial is one of the few studies to collect clinical health indicators beyond BW and anthropometrics in addition to questionnaires assessing health behaviors [40]. Even though there were no significant findings with regards to other indicators of health, the current study provides insights for future interventions.

While the findings of the current study do not support the *a priori* hypotheses, there are several explanations for these findings. Weight changes over the course of one year were expected to be relatively small, so the absence of a stronger effect of the nutrition education weight gain prevention intervention is not entirely surprising. As a majority of women in the RDG and CSG successfully prevented weight gain over one year, this is promising that nutrition education may play a role in weight gain prevention over the long term. Although participants did not significantly increase intakes of fiber, fruits, vegetables or whole grains over time, there were no appreciable adverse outcomes related to the biomarkers of health included in this study. Further, similar to Wong and colleagues [60] baseline indicators of health were not clinically abnormal in this population of women. Therefore, lifestyle and behavior changes made during the intervention period, while positive, may not have been large enough to produce a substantial impact [60]. Over time, these sustained lifestyle and behavior changes may result in more clinically meaningful improvements.

The lack of statistically significant findings also may be explained by the Hawthorne or observation effect [61]. Even though women in the CON group did not receive any intervention or information, their BW was assessed, along with a variety of other outcome measures at the same intervals as women receiving the nutrition education intervention. The Hawthorne effect may have resulted in CON participants modifying or altering behavior more so than under different circumstances, which may explain why there were no differences observed between the RDG and CSG compared to the CON. Additionally, women who enrolled in the study may have had more motivation to make lifestyle and behavior changes than individuals who were eligible and chose not to participate. Individuals who may benefit most from this type of intervention may not be adequately represented due to volunteer or self-selection bias [62].

Another explanation for non-significant findings may be the purposeful rotation of education leaders across education sessions. Providing exposure to multiple educators (i.e., four each) within the specified RDG or CSG group was done to specifically minimize potential effects of a particular educator on participant outcomes resulting from educator-participant bonding or external responsibility or support [63] rather than the intervention itself.

The inclusion of free-living women in this weight gain prevention intervention is a major strength of the current study, and findings from this study may be generalizable to similar populations of women with a desire to prevent weight gain. The nutrition education component of this intervention not only provided credible nutrition information, but also accountability and group support, all of which have been identified as facilitators to weight loss and weight loss maintenance [3]. Further, a majority of participants were compliant, indicating this was a feasible intervention with high participation. Although overall participant attrition was high at the conclusion of the intervention, nearly 75 % of the original sample remained through month 9 of the study, indicating that the intervention was realistic for free-living individuals.

This study is not without limitations. As previously mentioned, the final retention rate was moderate which limits statistical power of the study; thus, results should be interpreted with caution. However, a majority of individuals moved away from the area in the final month of the study and were unable to attend the final testing session. These “dropouts” were unique from women who chose to withdraw from the study for other reasons. While the use of free-living women was a strength, it was also a weakness as it is difficult to fully assess dietary intake and physical activity due to the limitations of self-report in addition to unmeasured and uncontrollable factors. Further, results from this study are only generalizable to women similar to those included in this study. More research is needed to target women of a wider range of ethnicities, education level and socioeconomic status. Future research should also examine weight gain prevention in men of this same age range, as well as postmenopausal women.

## Conclusions

In conclusion, no differences between women who received the weight gain prevention intervention and those randomized to a control group were found, as a majority of women were able to prevent weight gain during the study. These non-significant findings suggest that weight gain prevention over one year is possible; however, longer follow up periods are necessary. Clinically meaningful or significant benefits of participation in this study may become apparent over time, but long-term follow up data are not available. As weight loss and weight loss maintenance remain a challenge for much of the population, future interventions should emphasize indicators of health beyond BW and utilize a health promotion and weight gain prevention approach.

## Abbreviations

ANCOVA: analysis of covariance
BF%: body fat percentage
BMI: body mass index
BW: body weight
CON: control group
CSG: counselor group
FM: fat mass
GOAL: Groningen Overweight and Lifestyle
HDL-C: high-density lipoprotein cholesterol
IRB: Institutional Review Board
LDL-C: low-density lipoprotein cholesterol
MET: metabolic equivalent
RDG: registered dietitian group
TG: triglycerides

## References

[1] Flegal KM, Carroll MD, Kit BK, Ogden CL (2012). Prevalence of obesity and trends in the distribution of body mass index among US adults, 1999-2010. JAMA.

[2] Bacon L, Aphramor L (2011). Weight science: evaluating the evidence for a paradigm shift. Nutr J.

[3] Metzgar CJ, Preston AG, Miller DL, Nickols-Richardson SM (2015). Facilitators and barriers to weight loss and weight loss maintenance: a qualitative exploration. J Hum Nutr Diet.

[4] Mann T, Tomiyama J, Westling E, Lew AM, Samuels B, Chatman J (2007). Medicare’s search for effective obesity treatments: diets are not the answer. Am Psychol.

[5] Hill JO, Thompson H, Wyatt H (2005). Weight maintenance: what’s missing?. J Am Diet Assoc.

[6] Dansinger ML, Gleason JA, Griffith JL, Selker HP, Schaefer EJ (2005). Comparison of the Atkins, Ornish, Weight Watchers, and Zone diets for weight loss and heart disease risk reduction: a randomized trial. JAMA.

[7] Gardner CD, Kiazand A, Alhassan S (2007). Comparison of the Atkins, Zone, Ornish, and LEARN diets for change in weight and related risk factors among overweight premenopausal women: the A TO Z Weight Loss Study: a randomized trial. JAMA.

[8] Shai I, Schwarzfuchs D, Henkin Y (2008). Weight loss with a low-carbohydrate, Mediterranean, or low-fat diet. N Engl J Med.

[9] Makris A, Foster GD (2011). Dietary approaches to the treatment of obesity. Psychiatr Clin North Am.

[10] Sacks FM, Bray GA, Carey VJ (2009). Comparison of weight-loss diets with different compositions of fat, protein, and carbohydrates. N Engl J Med.

[11] McGuire MT, Wing RR, Klem ML, Seagle HM, Hill JO (1998). Long-term maintenance of weight loss: do people who lose weight through various weight loss methods use different behaviors to maintain their weight?. Int J Obes Relat Metab Disord.

[12] Wing RR, Phelan S (2005). Long-term weight loss maintenance. Am J Clin Nutr.

[13] Goodrick GK, Poston WS, Foreyt JP (1996). Methods for voluntary weight loss and control: update 1996. Nutrition.

[14] Jeffery RW, Drewnowski A, Epstein LH (2000). Long-term maintenance of weight loss: current status. Health Psychol.

[15] Byrne S, Cooper Z, Fairburn C (2003). Weight maintenance and relapse in obesity: a qualitative study. Int J Obes Relat Metab Disord.

[16] McGuire MT, Wing RR, Klem ML, Lang W, Hill JO (1999). What predicts weight regain in a group of successful weight losers?. J Consult Clin Psychol.

[17] Sjöström L, Lindroos AK, Peltonen M (2004). Lifestyle, diabetes, and cardiovascular risk factors 10 years after bariatric surgery. N Engl J Med.

[18] Christou NY, Look D, MacLean LD (2006). Weight gain after short- and long-limb gastric bypass in patients followed for longer than 10 years. Ann Surg.

[19] Peirson L, Fitzpatrick-Lewis D, Ali MU (2014). Prevention of overweight/obesity in adult populations: a systematic review with meta-analyses.

[20] van Genugten L, van Empelen P, Oenema A (2012). From weight management goals to action planning: identification of a logical sequence from goals to actions and underlying determinants. J Hum Nutr Diet.

[21] Bacon L, Stern J, Van Loan M, Keim N (2005). Size acceptance and intuitive eating improve health for obese, female chronic dieters. J Am Diet Assoc.

[22] Fagard RH (1999). Physical activity in the prevention and treatment of hypertension in the obese. Med Sci Sports Exerc.

[23] Appel LJ, Moore TJ, Obarzanek E (1997). A clinical trial of the effects of dietary patterns on blood pressure. N Engl J Med.

[24] Gaesser GA (2007). Exercise for prevention and treatment of cardiovascular disease, type 2 diabetes, and metabolic syndrome. Curr Diab Rep.

[25] Kraus WE, Houmard JA, Duscha BD (2002). Effects of the amount and intensity of exercise on plasma lipoproteins. N Engl J Med.

[26] Lamarche B, Despres JP, Pouliot MC (1992). Is body fat loss a determinant factor in the improvement of carbohydrate and lipid metabolism following aerobic exercise training in obese women?. Metabolism.

[27] Forster JL, Jeffery RW, Schmid TL, Kramer FM (1988). Preventing weight gain in adults: a pound of prevention. Health Psychol.

[28] Jeffery RW, French SA (1997). Preventing weight gain in adults: design, methods and one year results from the Pound of Prevention study. Int J Obes Relat Metab Disord.

[29] Jeffery RW, French SA (1999). Preventing weight gain in adults: the pound of prevention study. Am J Public Health.

[30] Levine MD, Klem ML, Kalarchian MA (2007). Weight gain prevention among women. Obesity (Silver Spring).

[31] ter Bogt NC, Bemelmans WJ, Beltman FW, Broer J, Smit AJ, van der Meer K (2009). Preventing weight gain: one-year results of a randomized lifestyle intervention. Am J Prev Med.

[32] ter Bogt NC, Milder IE, Bemelmans WJ (2011). Changes in lifestyle habits after counselling by nurse practitioners: 1-year results of the Groningen Overweight and Lifestyle study. Public Health Nutr.

[33] ter Bogt NC, Bemelmans WJ, Beltman FW, Broer J, Smit AJ, van der Meer K (2011). Preventing weight gain by lifestyle intervention in a general practice setting: three-year results of a randomized controlled trial. Arch Intern Med.

[34] Bennett GG, Foley P, Levine E (2013). Behavioral treatment for weight gain prevention among black women in primary care practice: a randomized clinical trial. JAMA Intern Med.

[35] Wing RR, Tate D, Espeland M (2013). Weight gain prevention in young adults: design of the study of novel approaches to weight gain prevention (SNAP) randomized controlled trial. BMC Public Health.

[36] Colvin RH, Olson SB (1983). A descriptive analysis of men and women who have lost significant weight and are highly successful at maintaining the loss. Addict Behav.

[37] Jeffery RW, Bjornson-Benson WM, Rosenthal BS, Lindquist RA, Kurth CL, Johnson SL (1984). Correlates of weight loss and maintenance over two years of follow-up among middle-aged men. Prev Med.

[38] Tiggerman M, Rothblum ED (1997). Gender differences in internal beliefs about weight and negative attitudes towards self and others. Pyschol Women Q.

[39] Stevens J, Truesdale KP, McClain JE, Cai J (2006). The definition of weight maintenance. Int J Obes (Lond).

[40] Metzgar CJ, Nickols-Richardson SM (2015). Determinants of weight gain prevention in young adult and midlife women: study design and protocol of a randomized controlled trial. JMIR Res Protoc.

[41] Zung WW (1965). A self-rating depression scale. Arch Gen Psychiatry.

[42] Piehowski KE, Preston AG, Miller DL, Nickols-Richardson SM (2011). A reduced-calorie dietary pattern including a daily sweet snack promotes body weight reduction and body composition improvements in premenopausal women who are overweight and obese: a pilot study. J Am Diet Assoc.

[43] Nickols-Richardson SM, Piehowski KE, Metzgar CJ, Miller DL, Preston AG (2014). Changes in body weight, blood pressure and selected metabolic biomarkers with an energy-restricted diet including twice daily sweet snacks and once daily sugar-free beverage. Nutr Res Pract.

[44] Knowler WC, Barrett-Connor E, Fowler SE (2002). Reduction in the incidence of type 2 diabetes with lifestyle intervention or metformin. N Engl J Med.

[45] Lutes LD, Winett RA, Barger SD (2008). Small changes in nutrition and physical activity promote weight loss and maintenance: 3-month evidence from the ASPIRE randomized trial. Ann Behav Med.

[46] Slawson DL, Fitzgerald N, Morgan KT (2013). Position of the Academy of Nutrition and Dietetics: the role of nutrition in health promotion and chronic disease prevention. J Acad Nutr Diet.

[47] Perri MG, Limacher MC, Durning PE (2008). Extended-care programs for weight management in rural communities: the treatment of obesity in underserved rural settings (TOURS) randomized trial. Arch Intern Med.

[48] Richardson MT, Ainsworth BE, Jacobs DR, Leon AS (2001). Validation of the Stanford 7-day recall to assess habitual physical activity. Ann Epidemiol.

[49] Friedewald WT, Levy RI, Fredrickson DS (1972). Estimation of the concentration of low-density lipoprotein cholesterol in plasma, without use of the preparative ultracentrifuge. Clin Chem.

[50] Hoaglin DC, Iglewicz B, Tukey JW (1986). Performance of some resistant rules for outlier labeling. J Am Stat Assoc.

[51] Hoaglin DC, Iglewicz B (1987). Fine tuning some resistant rules for outlier labeling. J Am Stat Assoc.

[52] Renjilian DA, Perri MG, Nezu AM, McKelvey WF, Shermer RL, Anton SD (2001). Individual versus group therapy for obesity: effects of matching participants to their treatment preferences. J Consult Clin Psychol.

[53] Dutton GR, Phillips JM, Kukkamalla M, Cherrington AL, Safford MM (2015). Pilot study evaluating the feasibility and initial outcomes of a primary care weight loss intervention with peer coaches. Diabetes Educ.

[54] Leahey TM, Wing RR (2013). A randomized controlled pilot study testing three types of health coaches for obesity treatment: professional, peer, and mentor. Obesity (Silver Spring).

[55] Katula JA, Vitolins MZ, Morgan TM (2013). The Healthy Living Partnerships to Prevent Diabetes study: 2-year outcomes of a randomized controlled trial. Am J Prev Med.

[56] West DS, Bursac Z, Cornell CE (2011). Lay health educators translate a weight-loss intervention in senior centers: a randomized controlled trial. Am J Prev Med.

[57] Parikh P, Simon EP, Fei K (2010). Results of a pilot diabetes prevention intervention in East Harlem, New York City: Project HEED. Am J Public Health.

[58] Durward CM, Hartman TJ, Nickols-Richardson SM (2012). All-cause mortality risk of metabolically healthy obese individuals in NHANES III. J Obes.

[59] Wildman RP, Muntner P, Reynolds K (2008). The obese without cardiometabolic risk factor clustering and the normal weight with cardiometabolic risk factor clustering: prevalence and correlates of 2 phenotypes among the US population (NHANES 1999-2004). Arch Intern Med.

[60] Wong MC, Wang HH, Kwan MW (2015). Dietary counselling has no effect on cardiovascular risk factors among Chinese grade 1 hypertensive patients: a randomized controlled trial. Eur Heart J.

[61] McCambridge J, Witton J, Elbourne DR (2014). Systematic review of the Hawthorne effect: new concepts are needed to study research participant effects. J Clin Epidemiol.

[62] Hedge MN (2007). A methodological review of randomized controlled trials. CDR.

[63] Melnyk B, Morrison-Beedy D (2012). Intervention research: design, conducting, analyzing and funding.

